# Antimicrobial Activities of Bacteria Associated with the Brown Alga *Padina pavonica*

**DOI:** 10.3389/fmicb.2016.01072

**Published:** 2016-07-12

**Authors:** Amel Ismail, Leila Ktari, Mehboob Ahmed, Henk Bolhuis, Abdellatif Boudabbous, Lucas J. Stal, Mariana Silvia Cretoiu, Monia El Bour

**Affiliations:** ^1^National Institute of Marine Sciences and TechnologiesSalammbô, Tunisia; ^2^Department of Marine Microbiology and Biogeochemistry, Royal Netherlands Institute for Sea Research and Utrecht UniversityYerseke, Netherlands; ^3^Department of Microbiology and Molecular Genetics, University of the PunjabLahore, Pakistan; ^4^Faculty of Mathematical, Physical and Natural Sciences of Tunis, Tunis El Manar UniversityTunis, Tunisia; ^5^Department of Aquatic Microbiology, Institute of Biodiversity and Ecosystem Dynamics, University of AmsterdamAmsterdam, Netherlands

**Keywords:** *Padina pavonica*, *Bacillus pumilus*, antibiotics, epibionts, seaweed

## Abstract

Macroalgae belonging to the genus *Padina* are known to produce antibacterial compounds that may inhibit growth of human- and animal pathogens. Hitherto, it was unclear whether this antibacterial activity is produced by the macroalga itself or by secondary metabolite producing epiphytic bacteria. Here we report antibacterial activities of epiphytic bacteria isolated from *Padina pavonica* (Peacocks tail) located on northern coast of Tunisia. Eighteen isolates were obtained in pure culture and tested for antimicrobial activities. Based on the 16S rRNA gene sequences the isolates were closely related to *Proteobacteria* (12 isolates; 2 Alpha- and 10 *Gammaproteobacteria*), *Firmicutes* (4 isolates) and *Actinobacteria* (2 isolates). The antimicrobial activity was assessed as inhibition of growth of 12 species of pathogenic bacteria (*Aeromonas salmonicida, A. hydrophila, Enterobacter xiangfangensis, Enterococcus faecium, Escherichia coli, Micrococcus* sp.*, Salmonella typhimurium, Staphylococcus aureus, Streptococcus* sp.*, Vibrio alginoliticus, V. proteolyticus, V. vulnificus*) and one pathogenic yeast (*Candida albicans*). Among the *Firmicutes*, isolate P8, which is closely related to *Bacillus pumilus*, displayed the largest spectrum of growth inhibition of the pathogenic bacteria tested. The results emphasize the potential use of *P. pavonica* associated antagonistic bacteria as producers of novel antibacterial compounds.

## Introduction

In aquatic environments, planktonic microbial communities live as free-living forms or attached to biotic or abiotic surfaces (Martin et al., 2014). In the marine environment, competition for space and nutrients is intense; the surfaces of marine eukaryotes such as seaweeds or invertebrates represent a suitable nutrient-rich habitat for microbial colonization and biofilm formation (Egan et al., 2008; Goecke et al., 2010). As the surfaces of theses eukaryotes are highly complex and differentiated, marine microbial biofilms should constitute a huge source of diversity, and the microbial communities forming them are expected to differ considerably in composition from pelagic microbial communities (Martin et al., 2014). Microbial community living on seaweed surfaces are highly complex, dynamic and consist of bacteria, fungi, diatoms, protozoa, spores and larvae of marine invertebrates (Lachnit et al., 2009, 2011; Goecke et al., 2010; Burke et al., 2011). Because especially bacteria interact with their host in multiple and complex ways, they constitute an interesting source of novel bioactive compounds with biotechnological potential (Martin et al., 2014). These bacteria may protect their host from harmful entities present in pelagic areas (Singh and Reddy, 2014) but can also produce compounds important for human and animal health (Kanagasabhapathy et al., 2006; Penesyan et al., 2010).

Bacteria may degrade algal polysaccharides such as for instance fucoidan (Nedashkovskaia et al., 2002) or alginates (Preston et al., 1986; Brown and Preston, 1991). Secondary metabolites are increasingly recognized for their importance for industrial and medical applications (Faulkner, 2000; Kelecom, 2002). Various novel compounds with antibiotic activity have already been identified from seaweed-associated bacteria (Chellaram et al., 2013; Horta et al., 2014; Martin et al., 2014). Several species of marine bacteria have been isolated from brown algae such as the spot-wounded fronds of *Laminaria japonica* (Sawabe et al., 2000), the surface of *Fucus serratus* (Johansen et al., 1999) and from rotten thallus of *Fucus evanescens* (Ivanova et al., 2004) and *Undaria pinnatifida* (Lee et al., 2006).

During summer, the brown alga *Padina pavonica* is abundantly present along the Tunisian coast. Despite its accessibility, nothing is known about the diversity and biological potential of its epibionts. Seaweeds of the genus *Padina* were previously reported to harbor epiphytic bacteria with variety of biological activities such as antibacterial activities of epiphytic bacteria isolated from *P. arborescens*, which was collected from Awaji Island, Japan (Kanagasabhapathy et al., 2006). Bacterial isolates from a close relative of *P. pavonica, P. tetrastromatica* collected from southeastern India were previously studied for their antibacterial activities against a panel of human pathogens (Chellaram et al., 2013; Ravisankar et al., 2013). However, since the geographic location is amongst the factors that impact on a bacterial community's diversity, composition and secondary metabolites production, the study of the epiphytic bacteria of *P. pavonica*, which is abundant at the Tunisian coast, was considered to be promising for finding novel antibacterial compounds.

The present study aimed at assessing the culturable bacterial diversity of the community attached to *P. pavonica*, by isolating and identifying the associated strains, and by testing their potential antimicrobial activity using a variety of pathogens as target organisms.

## Materials and methods

### Sampling

The seaweed *P. pavonica* (Class: Phaeophyceae, Order: Dictyotales, Family: Dictyotaceae) was collected during two seasons: winter and summer of 2007 from Cap Zebib (37° 16.2′N, 10° 3.6′E) at the northern coast of Tunisia. The algae were transferred in dark sterile plastic bags filled with water from the same location.

### Isolation of epiphytic bacteria

Seaweed samples were washed three times with autoclaved seawater in order to remove free-living and loosely attached bacteria (Burgess et al., 2003). Subsequently, the firmly attached epiphytic bacteria were extracted by vortexing 10 g of algal biomass in 90 ml autoclaved seawater for 6 min. Bacteria were isolated by serial dilution to 10^−3^ using autoclaved seawater. From each dilution 100 μl was spread-plated in triplicate on marine agar plates (MA: Pronadisa Laboratories, CONDA). The plates were incubated at 20°C until colonies appeared or at least for 7 days (Lemos et al., 1985). Visually distinct bacterial colonies were selected and further plated on MA until clonal cultures were obtained. The pure cultures were stored at −80°C in marine broth (Pronadisa Laboratories, CONDA) supplemented with 20% glycerol (v/v).

### Extraction of DNA and PCR conditions

Single colonies from the agar plates were suspended in sterile Milli-Q water and used as template in the PCR reactions using the bacterial 16S rRNA gene primer B8F and the universal primer U1492R (Lane, 1991; Table 1). PCR reactions were performed using a Gene Biometra T1 DNA Thermal Cycler (Perkin-Elmer Co. Norwalk-CT, USA) in 25 μl (final volume) reaction mixtures containing 0.1 μl of Hot Star Taq DNA polymerase (Qiagen), each primer at a final concentration of 10 pmol μl^−1^, each deoxynucleoside triphosphate at a concentration of 200 μM, 1.25 μl of 100% DMSO (Dimethylsulfoxide), 2.5 μl of BSA (Bovine Serum Albumin) at 0.2 mg ml^−1^ final concentration, 2.5 μl of PCR buffer (incl. MgCl_2_) and 1 μl of DNA template. The PCR protocol consisted of 40 cycles of denaturation at 94°C for 30 s, annealing at 55°C for 30 s and extension at 72°C for 1 min 50 s. The cycles were preceded by 15 min of denaturation at 94°C and ended with a final extension for 7 min at 72°C. Negative controls contained all components of the PCR mixture except the DNA template.

**Table 1:** **Primers used for PCR and DNA sequencing**.

**Primer**	**Sequence**
B8F	AGAGTTTGATCMTGGCTCAG
U1492R	GGTTACCTTGTTACGACTT
C5	AGAGTTTGATCCTGGCTCAGG
C26	GGGCGGTGTGTACAAGG
C72	CCGGAATIATTGGGCGTAA
C112	CTCGTTGCGGGACTTAACCC

### Analysis of PCR products

PCR products (2 μl) were analyzed by agarose gel electrophoresis (1% agarose, 1 × TAE buffer containing 40 mM Tris acetate and 1 mM EDTA, pH 8). Electrophoresis was performed at 100 V for 45 min. The gels were stained for 45 min with SYBR Gold nucleic acid gel stain (Invitrogen Corp.) and photographed under UV illumination.

### DNA sequencing

Approximately 100 ng DNA template was sequenced using the BigDye Terminator chemistry (Big Dye Terminator v3.1 Cycle Sequencing Kit, Applied Biosystems), according to the manufacturer's instructions. The sequence products were analyzed on an ABI 377 DNA sequencing system (Applied Biosystem) using the primers C5 (Gao et al., 2006), C26 (Ciancio et al., 2000), C72 (Gao et al., 2006), and C112 (Hauben et al., 1997; Table 1).

### Phylogenetic analysis

The closest phylogenetic relatives of each isolate were identified by comparison of the 16S rRNA gene sequence to the National Center for Biotechnology Information (NCBI) GenBank database using the Basic Local Alignment Search Tool (BLAST) analysis tools (http://www.ncbi.nlm.nih.gov/BLAST). Phylogenetic analysis of the sequences of the isolates and those of their selected top five best hit blast close relatives was performed using the Neighbor Joining method as available in MEGA version 4.1 software (Tamura et al., 2007).

### Nucleotide sequence accession numbers

Partial sequences of the 16S rRNA genes were submitted to GenBank and received the following accession numbers: P1 (FN652906), P2 (FN652907), P3 (FN652908), P4 (FN652909), P5 (FN652910), P6 (FN652911), P7 (FN652912), P8 (FN652913), P9 (FR695065), P11 (FR695066), P12 (FR695067), P14 (FR695068), P17 (FR695069), P18 (FR695072), P19 (FR695074), P20 (FR695070), P26 (FR695071), and P27 (FR695073).

### Antimicrobial activity

#### Microorganisms used for antimicrobial tests

Bacterial type strains (18 strains) used for the evaluation of antimicrobial activity of all isolated epiphytes (and also for alga extracts) were: *Aeromonas salmonicida* LMG3780*, A. hydrophila* B3 (RVAU-Denmark), *Enterococcus faecalis* ATCC 29212, *Escherichia coli* O126-B16 (ATTC 14948), *E. coli* ATCC 25922, *E. coli* ATCC 8739, *Micrococcus* sp. (Pasteur Institute, Tunis), *Pseudomonas cepacia* (INSTM, Tunis)*, P. fluorescens* AH2 (Danish Institute for Fisheries Research, Denmark), *P. aeruginosa* ATCC 27853, *Salmonella typhimurium* C52 (Laboratoire Hydrobiologie Marine et Continentale, Université de Montpellier II, France), *Staphylococcus aureus* (Pasteur Institute, Tunis)*, S. aureus* ATCC 25923, *S. aureus* ATCC 6538, *Streptococcus* sp. (Pasteur Institute, Tunis), *Vibrio tapetis* CECT4600 (Department of Microbiology and Parasitology, University of Santiago de Compostela, Spain), *V. anguillarum* ATCC12964T, *V. alginolyticus* ATCC 17749T. In addition the yeast *Candida albicans* ATCC 10231 was used for the tests.

The selected most active isolate against these 19 pathogens (the *Firmicutes* isolate P8) was also tested for antagonistic activity against nine *Vibrio* spp. type strains (pathogens well represented in marine ecosystems): *V. fluvialis* ATCC 33809T, *V*. (*Aliivibrio*) *logei* LMG14011, *V. parahaemolyticus* ATCC 17802 T, *V. pectenicida* CIP105190T, *V. pectenicida* LMG19642T, *V. proteolyticus* ATCC15338T*, V. salmonicida* LMG3780T, *V. splendidus* ATCC33125T, *V. vulnificus* ATCC27562T, and few other enteric bacteria species: *Enterobacter xiangfangensis* (CCY15226), *Enterococcus durans* (CCY15220), *Enterococcus faecium* (CCY15234), *Proteus mirabilis* (CCY15230), and *Morganella morganii* (CCY15221), originally obtained from the IFREMER Laboratory of Microbiology of Invertebrates (Brest, France).

[ATCC: American Type Culture Collection; CCY: Culture Collection Yerseke; LMG: Laboratory for Microbiology Gent; RVAU: Royal Veterinary and Agricultural University (now University of Copenhagen, Faculty of Life Sciences, Denmark); INSTM: National Institute of Sciences and Marine Technologies (Salammbô, Tunisia)].

#### Antimicrobial assay

Bacterial isolates were screened for their antimicrobial activity against human and fish pathogenic bacteria. The following two methods were used. The drop test assay on Trypto-Casein-Soy agar (TSA, BIO RAD) plates containing 20 g l^−1^ NaCl was carried out as described by James et al. (1996) and Rao et al. (2005) with slight modifications. Briefly, drops of cell suspension of an overnight culture were spotted onto the agar plates containing a confluent lawn of the target strain (dried for 30 min at 30°C) and then incubated at 30°C. The overlay assay was carried out as described by Wiese et al. (2009) with some modifications. Briefly, drops (10 μl) of an overnight culture of the isolates were spotted onto TSA plates supplemented with 20 g l^−1^ NaCl and incubated for 24 h at 30°C before they were covered with an overlay containing the target strains in soft agar [3 g l^−1^ Tryptone Soy Broth- (TSB-BIO RAD, 9 g l^−1^ agar (PRS, Panreac), 20 g l^−1^ NaCl, pH 7.2]. The suspension with the target strains contained ~10^7^ cells ml^−1^. Growth inhibition was evaluated after incubation (24 h at 30°C) by measuring the zone of inhibition around the spots with the test organism.

The antibacterial assay of algal crude extracts against its isolates was evaluated by disk diffusion method. Briefly, 500 μg of the total alga crude extract dissolved in dichloromethane and dichloromethane/methanol solvents (10 μl) was applied to sterile filter paper discs (6 mm). After solvent evaporation, the discs were placed on TSA plates, inoculated with an 18 h cultured associated strain (10^6^ bacteria. ml^−1^) in TSB containing 20 g. l^−1^ NaCl. As control, a disc loaded with solvent was simultaneously prepared. Plates were incubated overnight at 30°C. The diameter (in millimeter) of growth inhibition halo was measured after 24 h incubation considering the disc diameter. Assays were carried out in triplicate. These extracts were also tested in our previous work (Ismail-Ben Ali et al., 2010a) against the 18 indicator bacterial pathogens used in this study and cited above in material and methods.

### Preparing algal extracts

Fresh algae were dried during 15 days at room temperature in the dark and powdered. About 20 g of dried algae were extracted consecutively with two organic solvents with increasing polarity: dichloromethane (D) and dichloromethane/methanol (D/M) (1:1 v/v). Each extraction was carried out three times by maceration for 24 h at room temperature. The extracts were pooled, filtered, and concentrated under reduced pressure in a rotary evaporator. Algal extracts were stored at −20°C until use.

### Test of antibiotic sensitivity of isolated strains

The sensitivity to various antibiotics was investigated on Muller Hinton agar (MH, BIO RAD) plates by the disc diffusion method (Barry and Thornsberry, 1980), using the following antimicrobial compounds (BIO RAD, France), amounts are given per disc: streptomycin (500 μg), amoxicillin (25 μg), tobramycin (10 μg), nalidixic acid (30 μg) oleandomycin (15 μg), cefoxitin (30 μg), furans (300 μg), trimethoprim sulfamid (25 μg), penicillin G (6 μg), chloramphenicol (30 μg), tetracyclin (30 μg), oxacillin (1 μg), ceftriaxon (30 μg). A strain was considered susceptible to an antimicrobial compound if any growth inhibition zone was observed around the disc. Interpretation was given according the guidelines of the French Society for Microbiology (Soussy, 2008).

### Extracellular activity test

#### Preparation of the cell-free supernatant

Ten ml of culture of the isolates P9, P11, P12, and P20 grown for 48 h at 30°C in TSB was centrifuged at 5000 rpm for 15 min and the supernatants were transferred to new tubes. Activities of the cell-free supernatants were evaluated by the drop method on TSA plates previously inoculated with *Micrococcus* sp., *A. salmonicida* LMG3780T or *S. aureus*. The same protocol was used for isolate P8 using cultures taken after 24, 48, and 72 h of growth.

#### Effects of enzymatic and heat treatments on antibacterial activity

##### Protease effect

The enzymatic effect on the antibacterial activity was evaluated on cell-free supernatants of the active isolates. Samples of 1 ml were treated at 37°C for 2 h with 1 mg ml^−1^ final concentration of trypsin and pepsin. The residual activities of the samples were assayed by the drop method.

##### Heat effect

To analyze thermal stability, supernatants were heated to 60°C for 30 min, 80°C for 10 min, and 100°C for 5 min and then cooled to room temperature before being tested for antibacterial activity.

## Results

### Alga epiphytic bacteria identification

Epiphytic bacteria were extracted from *P. pavonica* thallus and plated on MA. A total number of 26 isolates was obtained after the first round of cultivation of algal samples. Colony morphology dereplication resulted in 18 bacterial isolates that were obtained in pure culture. Analysis of the 16S rRNA gene sequences showed that all isolates were affiliated to three major groups within the bacterial domain, *Alpha-* and *Gammaproteobacteria, Firmicutes* and *Actinobacteria* (Table 2). Most of the isolates belongs to the proteobacterial group (twelve isolates: P1–P5, P9, P11, P12, P14, P17, P20, and P26). Two isolates belong to *Alphaproteobacteria*: *Paracoccus* (P1) and *Devosia* (P2) and ten isolates affiliated with the *Gammaproteobacteria* which were closely related to *Pseudoalteromonas* sp. (P3, P12, and P14), *Pseudomonas* sp. (P9, P11 and P20), *Pseudomonas putida* (P5), *Acinetobacter* (P17), *Erwinia billingiae* (P26) and *Vibrio* sp. (P4). The phylum of the Gram-positive *Firmicutes* was represented by four isolates closely related to *Bacillus pumilus* (P8), *Planomicrobium glaciei* (P18), and *Staphylococcus* sp. (P7 and P27). Isolate P27 is related to *Staphylococcus* but with less than 90% identity. Two isolates (P6 and P19) were closely related to the genus *Brevibacterium* of the phylum *Actinobacteria*.

**Table 2:** **Identification of 18 bacterial strains associated with ***Padina pavonica*** based on 16S rRNA gene sequence identity obtained by BLAST analysis**.

**Isolate**	**Accession number**	**Best hit**	**Identity (%)**	**Phylum**
P1	FN652906	*Paracoccus* sp.	98	Alphaproteobacteria
P2	FN652907	*Devosia* sp.	99	Alphaproteobacteria
P3	FN652908	*Pseudoalteromonas* sp.	99	Gammaproteobacteria
P4	FN652909	*Vibrio* sp.	96	Gammaproteobacteria
P5	FN652910	*Pseudomonas putida*	98	Gammaproteobacteria
P6	FN652911	*Brevibacterium iodinum*	96	Actinobacteria
P7	FN652912	*Staphylococcus* sp.	97	Firmicutes
P8	FN652913	*Bacillus pumilus*	99	Firmicutes
P9	FR695065	*Pseudomonas* sp.	93	Gammaproteobacteria
P11	FR695066	*Pseudomonas* sp.	95	Gammaproteobacteria
P12	FR695067	*Pseudoalteromonas* sp.	100	Gammaproteobacteria
P14	FR695068	*Pseudoalteromonas* sp.	94	Gammaproteobacteria
P17	FR695069	*Acinetobacter* sp.	95	Gammaproteobacteria
P18	FR695072	*Planomicrobium glaciei*	100	Firmicutes
P19	FR695074	*Brevibacterium* sp.	98	Actinobacteria
P20	FR695070	*Pseudomonas* sp.	100	Gammaproteobacteria
P26	FR695071	*Erwinia billingiae*	99	Gammaproteobacteria
P27	FR695073	*Staphylococcus* sp.	88	Firmicutes

### Host and epibionts affinity

To verify the close relationship between host and isolated bacteria, antibacterial activity of *P. pavonica* organic crude extracts (polar and non-polar) were tested against the 18 isolates. The results revealed that extracts of the alga harvested in summer exhibited a weak activity (1 mm diameter) against three of the 18 isolates, P6, P7, and P8. While the same extracts had a pronounced inhibitory activity against human and animal pathogens indicator bacteria (9 amongst 18 were inhibited) (Ismail-Ben Ali et al., 2010a; **Figure 4**).

### Epiphytic bacteria potential activity

**Figure 1:**
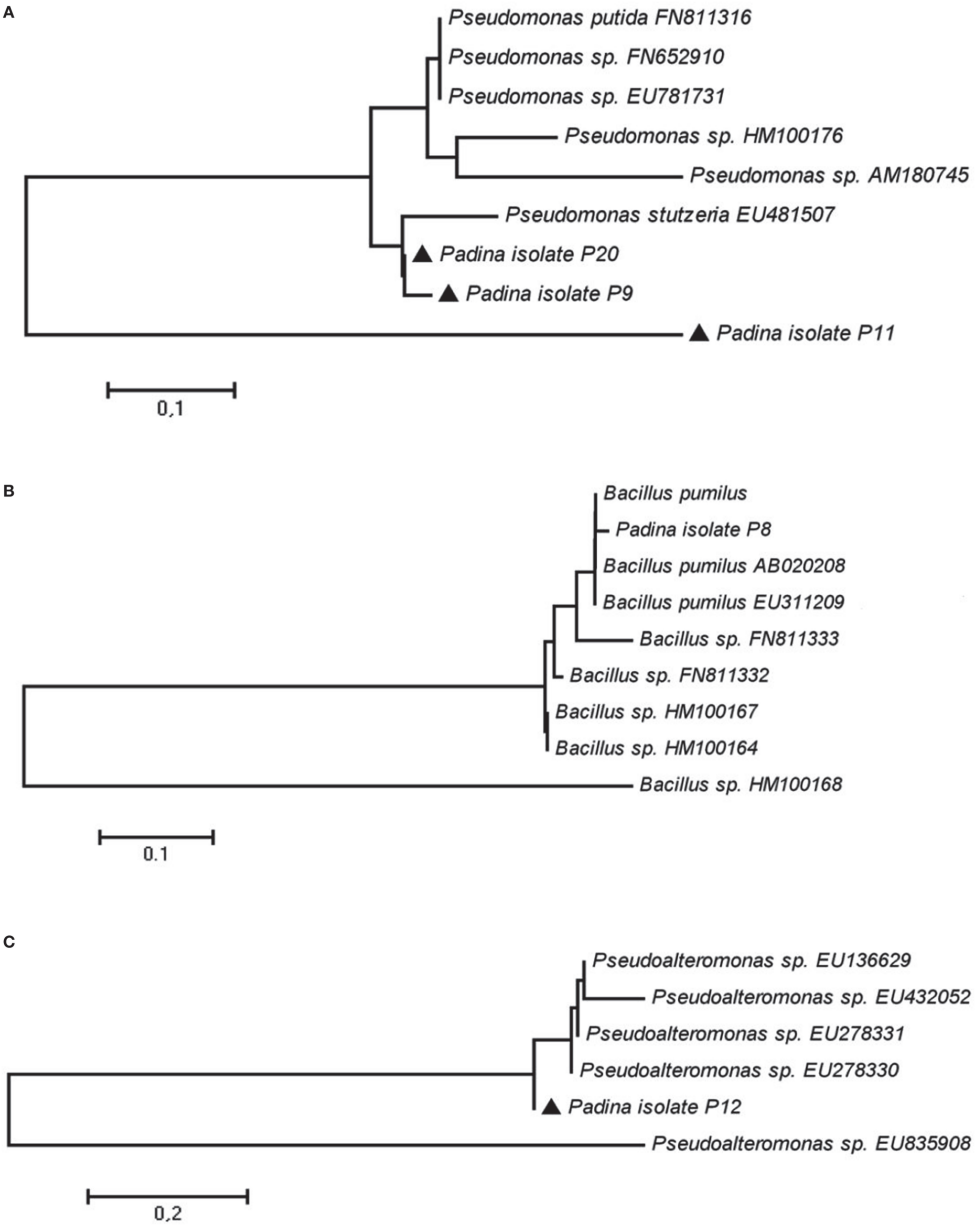
**Neighbor-joining tree based on 16S rRNA gene sequences derived from ***P. pavonica*** isolates and top five blast best hit sequences identified via GenBank search. (A)** P9, P11, and P20 isolates and *Pseudomonas* genus **(B)** P8 isolate and *Bacillus* genus **(C)** P12 isolate and *Pseudoalteromonas* genus.

The 18 isolates were tested for their antimicrobial activities using two different methods and a number of selected pathogens. Five isolates: P8, P9, P11, P12, and P20 showed antibacterial activities against a broad range of pathogens (Table 3). Phylogenetic analysis of the 16S rRNA gene sequences of these active isolates was performed using the Neighbor Joining method (Figure 1).

**Table 3:** **Antibacterial and antifungal activities of isolate P8, P9, P11, P12, and P20 against pathogen bacteria**.

**Test isolates**	**P8**		**P9**		**P11**		**P12**		**P20**	
	**D**	**O**	**D**	**O**	**D**	**O**	**D**	**O**	**D**	**O**
*E. coli* O126B16 ATTC 14948	+	++	−	−	−	−	−	−	−	−
*E. coli* ATCC 25922	++	+++	−	−	−	−	−	−	−	−
*E. coli* ATCC 8739	++	+++	−	−	−	−	−	−	−	−
*V. tapetis* CECT4600	+	+	+	++	+	++	+	++	+	++
*V. anguillarum*	−	−	−	−	−	−	−	−	−	−
*V. alginoliticus*	+	+++	−	−	−	−	−	−	−	−
*P. cepacia* (INSTM)	−	−	−	−	−	−	−	−	−	−
*P. fluorescens* AH2	+	++	−	−	−	−	−	−	−	−
*P. aeruginosa* ATCC 27853	−	−	−	−	−	−	−	−	−	−
*A. salmonicida* LMG3780	++	++++	−	−	−	−	−	−	−	−
*A. hydrophila* B3	+	+++	+	++	+	+	+	+	+	+
*S. typhymurium* C52	+	+++	++	++	++	++	++	++	++	+++
*Streptococcus* sp. (Pasteur Inst)	+	+++	+	+	+	+	+	+	+	++
*S. aureus (Pasteur Inst)*	+++	++++	+++	+++	+++	+++	+++	+++	+++	+++
*S. aureus* ATCC 25923	+	++	+	++	+	++	+	++	+	++
*S. aureus* ATCC 6538	++	++++	+	+	+	+	+	+	+	+
*E. faecalis* ATCC 29212	+	++	+	+	+	+	+	+	+	+
*Micrococcus* sp.(Pasteur Inst)	++	+++	++	++	++	++	++	++	++	+++
*C. albicans* ATCC 10231	++	+++	−	−	−	−	−	−	−	−

*D, Drop method; O, overlay method; Inhibition zones > to 25 mm were declared as very strong (++++), Inhibition zones from 15 to 25 mm as strong (+++), from 7 to 15 as moderate (++) and < 7 as weak activities (+), no activity (−)*.

As shown in Table 3, isolates P9, P11, P12, and P20 had the same antibacterial spectrum and were effective against nine indicator strains tested. The antibiotic properties were most effective against the pathogens *S. aureus, S. typhymurium*, and *Micrococcus* sp. Growth of the yeast *C. albicans* was not inhibited by these isolates. Isolate P8 (related to *Bacillus pumilus*) displayed the strongest antimicrobial activity and was effective against 15 tested bacteria and the yeast *C. albicans*. A weak inhibition of P8 was observed against *V. tapetis* and no inhibition of *V. anguillarum, P. cepacia*, and *P. aeruginosa* was observed.

**Figure 2:**
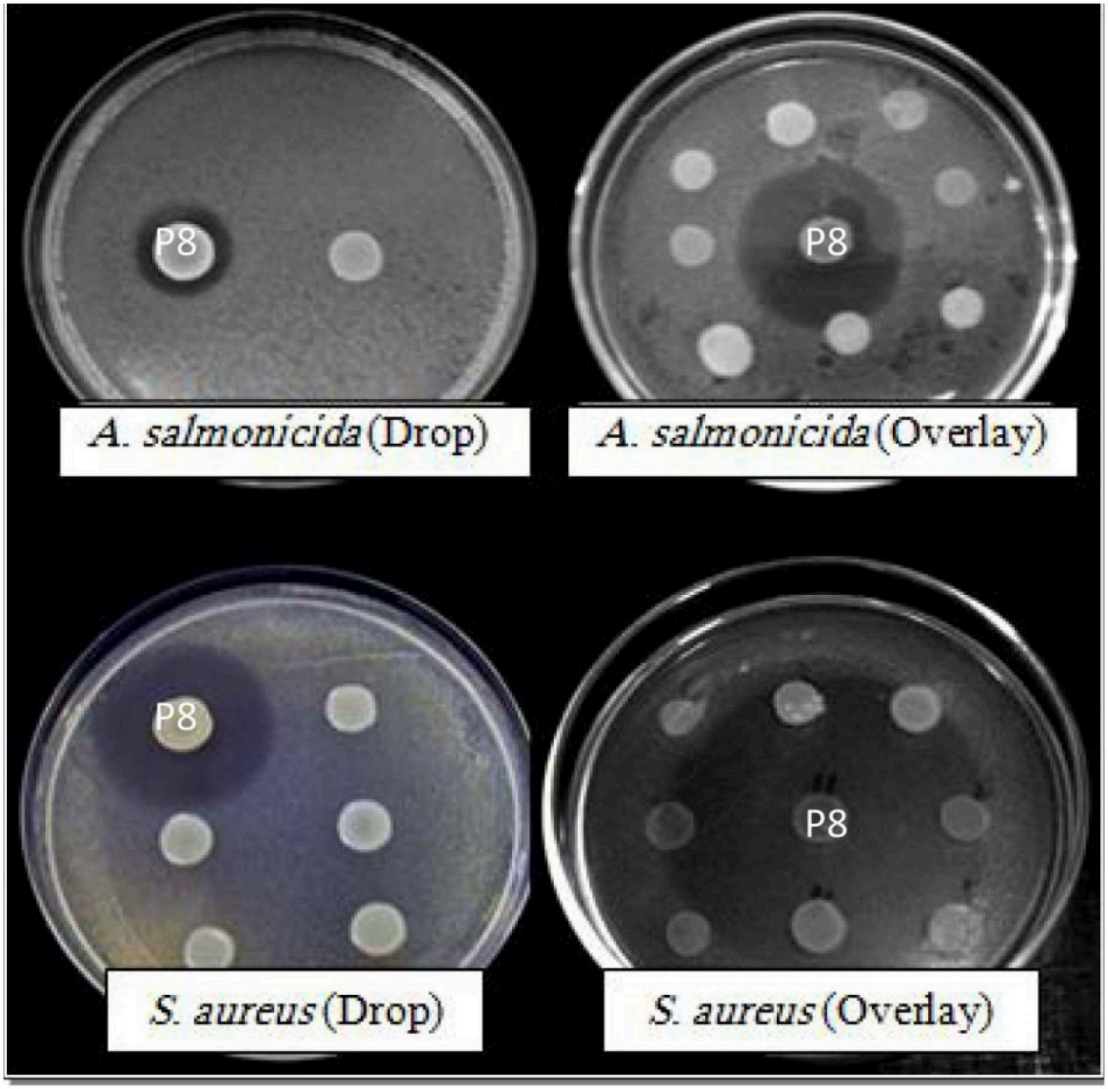
**Plates testing antimicrobial activity of strain P8 isolated from ***P. pavonica*** by using the drop method and the overlay assay against ***A. salmonicida*** and ***Staphylococcus aureus*****.

Inhibition zones depended on the method applied. Generally larger inhibition zones were observed when using the overlay assays compared to the drop assay (Table 3). The strongest effect was recorded for isolate P8 against *S. aureus* (inhibition zones varied from 20 to 45 mm; Figure 2). On the basis of its strong activity, isolate P8 was selected to test its antagonistic activity against nine *Vibrio* species, which are well known marine (fish, shellfish) pathogens. The overlay method was chosen for this test, showing a higher effectiveness when compared to the drop method. As shown in Table 4, two strains of *Vibrio* species tested were inhibited (*V. proteolyticus* and *V. vulnificus*) and furthermore *E. xiangfangensis* and *E. faecium* were inhibited as well.

**Table 4:** **Antagonistic activity of isolate P8 against sensitive ***Vibrio*** spp. and other bacteria using the overlay method (Wiese et al., 2009)**.

**Strain**	**Inhibition**
*V. proteolyticus*	+++
*V. vulnificus* ATCC 27562 T	+
*E. xiangfangensis*	++
*E. faecium*	+++

*Inhibition zones from 15 to 25 mm were considered strong (+++), from 7 to 15 moderate (++) and < 7 weak activities (+)*.

### Test of antibiotic sensitivity of isolated strains

The five isolates with clear antimicrobial activity were further characterized. Their own sensitivity against various antibiotics was investigated. Isolate P8 showed resistance to cefoxitin, oxacillin, and ceftriaxon. P9, P11, and P20 were resistant to amoxicillin, cefoxitin, streptomycin, tetracycline, penicillin G, oxacillin, furans, and ceftriaxone. P11 was also resistant to chloramphenicol. P12 was resistant to amoxicillin, cefoxitin, streptomycin, tobramycin, penicillin G and oxacillin.

### Effects of enzymatic and heat treatments on antibacterial activity

The cell-free supernatants of cultures of the five active isolates were tested for presence of antimicrobial activity against *Micrococcus* sp. and *S. aureus* (the most sensitive species retained). Supernatants of cultures of the 5 isolates inhibited growth of both test strains (Table 5). The effect of incubation time on the production of active compounds was tested in isolate P8 after 24, 48, and 72 h of incubation. Antibacterial activity was detected for all three incubation periods, with the strongest activity after 48 h incubation, compared by the activity detected after 24 and 72 h.

**Table 5:** **Effects of enzymatic and heat treatments on antibacterial activity of ***Padina pavonica*** active isolates supernatant**.

	***Micrococcus*** **sp**.						***S. aureus***					
	**S**	**T**	**P**	**T1**	**T2**	**T3**	**S**	**T**	**P**	**T1**	**T2**	**T3**
P8	+	+	+	+	+	−	+	+	+	+	+	−
P9	+	+	+	−	−	−	+	+	+	−	−	−
P11	+	+	+	−	−	−	+	+	+	−	−	−
P12	+	+	+	−	−	−	+	+	+	−	−	−
P20	+	+	+	−	−	−	+	+	+	−	−	−

*S, supernatant; T, supernatant treated by trypsin; P, supernatant treated by pepsin; T1, supernatant heated at 60°C; T2, supernatant heated at 80°C; T3, supernatant heated at 100°C. +, active; −, not active*.

Results of the sensitivity tests to proteases and heat treatment (Table 5) showed that bioactive substances produced by isolates P8, P9, P11, P12, and P20 were insensitive to trypsin and pepsin treatments. However, the bioactive substances were thermolabile. Antibacterial activity for isolates P9, P11, P12, and P20, was totally lost after being heated at 60°C for 30 min.

**Figure 3:**
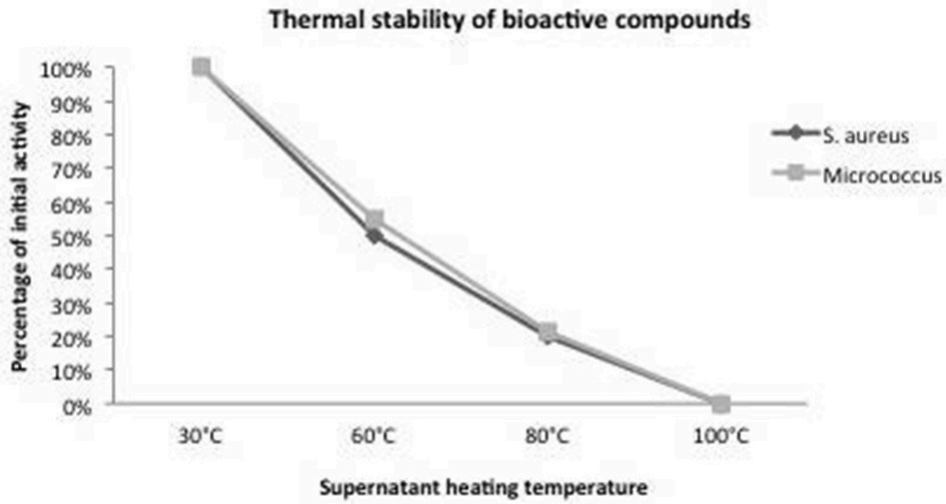
**Influence of supernatant heating treatment on the P8 antibacterial activity against ***Staphylococcus aureus*** and ***Micrococcus*** sp**.

The active substance produced by isolate P8 was more resistant to increased temperatures since activity was not completely lost at 60°C but decreased with increasing temperature. About 50% of the diffusion zone remained after incubation at 60°C and 20% at 80°C, while the activity was totally lost after a treatment for 5 min at 100°C (Figure 3).

## Discussion

The brown alga *P. pavonica* is widespread along the Tunisian coastline, especially in the northern part (Cap Zebib). This alga can be found up to 10 m depth with a total estimated biomass of 112.5 tons of fresh matter (25 tons dry matter; Ksouri et al., 2008). A previous study by Ktari and Guyot (1999) highlighted the cytotoxic activity of a dichloromethane extract of *P. pavonica* against KERATIN-forming tumor cell line HeLa (KB cells). Besides, Omezzine et al. (2009), reported antifungal potential of this alga collected from Tunisian coast.

Several studies reported interactions between brown algae and their epibionts (Lee et al., 2006). Some brown algae-associated bacteria may degrade algal polysaccharides, such as fucoidan (Nedashkovskaia et al., 2002), or alginates (Preston et al., 1986; Brown and Preston, 1991). Brown algae may produce biologically active compounds that are capable of killing bacteria (bactericidal) or inhibiting bacterial growth (bacteriostatic; Nagayama et al., 2002).

In this study, a number of 26 isolates were firstly extracted from *P. pavonica* thallus at a first round cultivation on MA, then restricted to 18 after subculturing to obtain pure morphological different colonies since algal samples were subjected to a harsh pre-treatment in order to be sure that only attached bacteria will be isolated. Also in other previous study by Kanagasabhapathy et al. (2006), numbers of culturable isolates from some brown algae collected from Awaji Island Japan; were 10 for *Padina arborescens* and 21, 19, 17, 15, 12, 9, 8, and 5 for *Ecklonia cava, Colpemenia sinusa, Sargassum serratifolium, S. fusiforme, Petalonia fascia, Scytosiphon lomentaria, S. filicinum*, and *Undaria pinnatifida* respectively.

In other previous study by Albakosh et al. (2016) on the brown alga *Splachnidium rugosum*, a number of 41 isolates were obtained using 4 different medium; MA, nutrient sea water agar, nutrient agar and thiosulfate-citrate-bile-salts-sucrose agar. In this same study by Albakosh et al. (2016), initially 29 isolates were obtained on MA medium and after identification, the number was narrowed down to 22 unique isolates. From our results and these previous work and observations it seems that MA is a suitable medium to isolates algal epiphytic bacteria as compared to other medium. However, the number of culturable epiphytic bacteria will increase when using different culturing media.

After characterization, Gram-negative *Alpha*- and *Gammaproteobacteria* and Gram-positive *Firmicutes* and *Actinobacteria* were identified from *P. pavonica*. Most of the isolates belonged to Gram-negative bacteria, as was the case for epibionts of seaweeds (Lakshmanaperumalsamy and Purushothaman, 1982; Albakosh et al., 2016). Among the different *P. pavonica* isolates, isolates related to the genera *Paracoccus, Acinetobacter*, and *Staphylococcus* were reported in previous studies isolated from marine environments and seaweeds (Gallardo et al., 2004; Schulze et al., 2006; Li, 2009; Zeng et al., 2010). Bacteria of the genera *Planomicrobium* were previously isolated from seawater and sediment and have algicidal effect, affecting harmful algal blooms (Giudice et al., 2007; Zeng et al., 2010). The actinobacterial genus *Brevibacterium* (P6, P19) is found in marine and terrestrial environments (Jones and Keddie, 1953; Collins, 1992) and were previously also isolated from degraded thallus of the brown alga *Fucus evanescens* (Ivanova et al., 2004). Two of the *P. pavonica* associated isolates reported in this study, P2 and P26, closely related to the *Proteobacteria*: *Devosia* and *Erwinia*, respectively, were previously not known from the surfaces of marine seaweeds. Species of the genus *Devosia* have been isolated from beach sediment (Lee, 2007), from freshwater legume *Neptunia natans* (Rivas et al., 2003), as endosymbionts of the marine ciliate *Euplotes magnicirratus* (Vannini et al., 2004), from a nitrifying inoculum (Vanparys et al., 2005) and from soil and greenhouse soil (Yoo et al., 2006; Yoon et al., 2007). Bacteria of the genus *Erwinia* are known as plant pathogens (Paulert et al., 2007) and common inhabitants of the rhizosphere of cultivated crops and weeds (Burr and Schroth, 1977). Besides, in this study we notice that P9, P11, P14, P17, and P27 isolates may be considered as potentially novel species since they were related with less than 95% identity to *Pseudomonas* sp. (P9, P11), *Pseudoalteromonas* sp. (P14), and *Acinetobacter* sp. (P17). Isolate P27 may be a potentially novel genus, since it is less than 90% identical with its closest relative *Staphylococcus*. These isolates may be of biotechnological interest and need further characterization.

**Figure 4:**
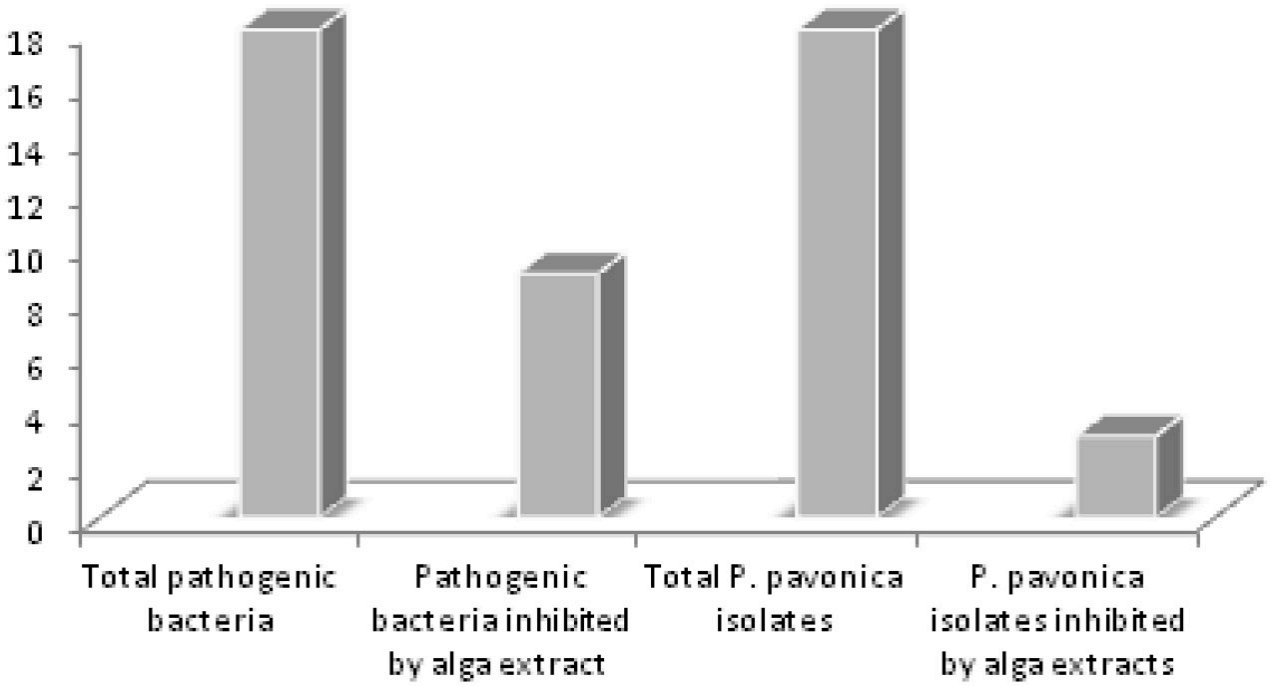
**Comparison between ***P. pavonica*** crude extracts inhibition spectrum against sensitive alga isolates and pathogenic human and fish sensitive bacteria (18 pathogens)**.

*P. pavonica* harbors several culturable epiphytic bacteria on its surface and our harsh isolation procedure indicates that these epibionts are intimately associated with their host. We tested the resistance of the isolates to crude extracts of the *P. pavonica*. Only extracts from *P. pavonica* harvested in summer revealed a low inhibitory effect toward three of the isolates. Organic crude extracts of *P. pavonica* were toxic against human and fish pathogens such as *V. tapetis, V. anguillarum, V. alginoliticus, Ps. cepacia, A. salmonicida, A. hydrophila, E. faecalis* ATCC 29212, and *S. aureus* (Ismail-Ben Ali et al., 2010a). In Figure 4 a diagram is depicted showing the number of pathogenic bacteria inhibited by *P. pavonica* extracts, which represent 50% of the total number of pathogens tested (Ismail-Ben Ali et al., 2010a), compared to *P. pavonica* isolates that are inhibited by their host extracts, which represent only 16.6% of the total isolates tested. Chbani et al. (2011) and Zerrin et al. (2011) also reported toxicity of *P. pavonica* against pathogenic indicator bacteria.

In this study 5 of the 18 isolates possessed antimicrobial activities. In our previous work we isolated 10 bacteria from the green alga *U. intestinalis* with two strains showing antimicrobial potential and 19 from *J. rubens* with 7 strains displaying antibacterial activity against pathogenic indicator bacteria (Ismail-Ben Ali et al., 2010b, 2012).

Many *Pseudoalteromonas* species are associated with eukaryotic hosts in the marine environment (Holmström and Kjelleberg, 1999). Such species have been isolated from a variety of animals, such as mussels, tunicates and sponges, as well as from a range of marine plants (Holmström and Kjelleberg, 1999) and from the red alga *Jania rubens* collected from Tunisian coast (Ismail-Ben Ali et al., 2012). These *Pseudoalteromonas* species displayed different extracellular biological activities ranging from antibacterial, agarolytic, antiviral, antifouling, to algicidal activities (Sawabe et al., 1998; Holmström and Kjelleberg, 1999; Yuexin et al., 2010). Pseudoalteromonads may play an important role in the control of harmful algal blooms within the marine environment by the production of an extracellular inhibitor that causes rapid lyses of algal species within the genera *Chattonella, Gymnodinium*, and *Heterosigma* (Lovejoy et al., 1998). *Pseudomonas, Pseudoalteromonas* and *Vibrio* species have also been isolated from the brown alga *Laminaria saccharina* and possess antibacterial substances (Wiese et al., 2009). Previously, we isolated *Pseudoalteromonas, Pseudomonas* and *B. pumilus* species from the surface of the red alga *J. rubens* collected from the Tunisian coast. These isolates showed a varying spectrum of antimicrobial activity (Ismail-Ben Ali et al., 2012). *Vibrio* sp.*, Pseudomonas* sp. and *B. pumilus* are known as probiotic bacteria used in aquaculture (Hill et al., 2009). Epiphytic *Vibrio* strains produced various antibacterial compounds (Oclarit et al., 1994). However, in our study 13 strains of bacteria that were isolated from the surface of *P. pavonica* did not show any antibacterial activity. These isolates may have another ecological function. For instance, they may have a protective role by releasing compounds into the surrounding seawater that would help preventing extensive fouling of the surface (Burgess et al., 1999; Kanagasabhapathy et al., 2008).

Bacteria of the genus *Bacillus*, to which our isolate strain P8 belongs, have been reported to be associated with various marine organisms including brown algae (Thakur and Anil, 2000). Lee et al. (2006) described *B. psychrodurans* as a representative strain associated with the brown alga *Undaria pinnatifida*. *B. pumilus* was isolated by Wiese et al. (2009) from the surfaces of the brown alga *L. saccharina* as well as from the surface of other brown algae such as *Sargassum fusiforme, Colpomenia sinuosa*, and *Ecklonia cava* (Kanagasabhapathy et al., 2006). Similarly, Parvathi et al. (2009) isolated *B. pumilus* strains from sediment, crab, fish, starfish and oyster. In the Table 6 the main studies on *B. pumilus* isolated from algae and other sources are summarized and the biological activities and compounds responsible for these inhibitory activities are reported. *Bacillus* is well known for producing antimicrobial compounds (Wiese et al., 2009). Since 1955, *B. pumilus* was described as antibiotic producer; Pumilin is an antibiotic isolated from this bacterial strain by Bhate (1955) and it is mainly active against gram positive microorganisms. Various metabolites with antibiotic activity, including the important group of antibiotic peptides like polymixin B, gramicidin and bacitracin are produced by *Bacillus* species (Crisley, 1964; Vandamme and Demain, 1976; Ishihara et al., 2002). Results obtained in this study are in agreement with these reports. P8 bacteria showed a broad inhibition spectrum against human and fish pathogens and the strongest inhibitory activity was observed against *S. aureus* and *A. salmonicida. Bacillus* sp. isolated from seaweeds and sediment by Suvega and Arunkumar (2014) had strong activities against bacteria plant pathogens (*Xanthomonas axonopodis* pv. *citri, X. oryzae* pv. *oryzae*) and fungi (*Ustilaginoidea virens*) causing diseases in rice paddy fields.

**Table 6:** **Biological activities of ***Bacillus pumilus*** isolated from marine algae and other sources**.

**Origin**	**Inhibition of microorganisms/applications**	**References**
*Penaeus monodon* (black tiger shrimp)	Marine bacterial pathogens *Vibrio alginolyticus, V. mimicus* and *V. harveyi*	Hill et al., 2009
	*B. pumilus* used as probiotic in aquaculture.	
*Ulva* spp. (green alga)	*S. aureus, E. coli* and *S. Typhimurium*	Prieto et al., 2012
*Fucus vesiculosus* (brown alga)		
*Polysiphonia lanosa* (red alga)		
*Palmaria palmata* (red alga)		
*Fucus serratus* (brown alga)		
*Colpomenia sinuosa* (brown alga)	Gram positive and Gram negative fouling bacteria; *Vibrio* and *Photobacterium* species: (*V. fischeri V. harveyi V. alginolyticus P. phosphoreum P. damselae)* and pathogens of the genus: *Staphylococcus, Serratia, Klebsiella* and *Salmonella*	Kanagasabhapathy et al., 2006
*Jania rubens* (red alga)	*Pseudomonas aeruginosa, Aeromonas salmonicida, A. hydrophila, Salmonella typhimurium, Streptococcus* sp*., S. aureus, Enterococcus feacalis, Micrococcus* sp*., Candida albicans*.	Ismail-Ben Ali et al., 2012
Soil	*Micrococcus luteus* ATCC 10240 and *S. aureus* ATCC 6538	Hasan et al., 2009
Soil	Bacitracin (peptide) active against *Micrococcus luteus* and *S. aureus*	Awais et al., 2007
Endophytic bacteria from ethnovarieties of cassava	Active against the fungi *Rhizoctonia solani, Pythium aphanidermatum* and *Sclerotium rolfsii*. The antifungal compound was identified as pumilacidin	Pereira de Melo et al., 2009
Lipopeptide biosurfactants (LPBSs) from *Bacillus* sp	Agriculture, chemical, food, and pharmaceutical industries	Roongsawang et al., 2010
Soil and water samples	Pumilicin 4, inhibits several Gram-positive bacteria, including MRSA and VRE	Aunpad and Na-Bangchang, 2007; Abriouel et al., 2011
Soil	*E. coli* ATCC 2939, *Staphylococcus aureus* ATCC 96, *Bacillus subtilis* ATCC 441, *Aspergillus niger* A1781 and *Aspergillus flavous* A873	Sawale et al., 2014
Clinical specimens	Fungicidal activity against Mucoraceae and *Aspergillus* spp	Bottone and Peluso, 2003
Soil	*S. aureus, S. pyogens, Klebsiella spp., S. typhi*	Kuta et al., 2009

*MRSA, Methicillin-Resistant Staphylococcus aureus; VRE, Vancomycin-Resistant-Enterococci; ATCC, American Type Culture Collection*.

In our previous study (Ismail-Ben Ali et al., 2012) a *B. pumilus* related strain was isolated from the red alga *J. rubens* and had a different antibacterial spectrum than the isolate from *P. pavonica*, confirming that the algal species not only determines epiphytic diversity but also affects the type of secondary metabolite that is produced, even among the same species of epiphyte.

Kumar et al. (2014) reported antibacterial activities cell free supernatant of a culture of a *Bacillus* strain isolated from entomopathogenic nematode against *E. coli*. Only a few epiphytic bacteria isolated from algae are known to have inhibitory potential against the human pathogen *E. coli*. Inhibition of *E. coli* by isolates, P9, P11, P12, and P20 and especially by P8 suggests that these isolates may produce novel antimicrobial substances. P8 inhibits both Gram+ and Gram- pathogens. This finding is supported by other studies such as those of Chatterjee et al. (2008) and Prieto et al. (2012) who reported antibacterial activities of *Bacillus* strains isolated from reef sediments and marine alga against *P. aeruginosa, S. aureus, Salmonella enterica, Bacillus* sp., *Proteus vulgaris, E. coli*, and *S. typhimurium*. Gram negative isolates such as *Pseudomonas* and *Pseudoalteromonas* strains (P9, P10, P11, and P20) have variable antibacterial activities toward both Gram+ and Gram− tested pathogens. Qi et al. (2009) and Horta et al. (2014) reported Gram− marine isolates (from *Bifurcaria bifurcata* and deep see sediment) such as *P. rhizosphaerae, Vibrio* sp., *Alteromonas* sp., *Shewanella* sp., and *Serratia* sp. active against Gram+ pathogens.

Antimicrobial tests were carried out by two different methods, the drop technique and the overlay assay. The overlay assay was better suitable for the detection of antibacterial and antifungal activity of bacterial isolates. Spots of isolates that are grown overnight may already have secreted bioactive compounds in the agar before they were overlaid by the target strain. Therefore, it was concluded that the five active isolates closely related to *Bacillus, Pseudomonas* and *Pseudoalteromonas*, produced and excreted bioactive(s) inhibitory substance(s) during 24 h growth. When these organisms were dropped simultaneously with the target organism the zone of inhibition was much less apparent. Perhaps the target organism prevented growth of the test organism or its production or excretion of the bioactive compound.

Inhibitory compounds produced by the five active isolates were present in the cell-free spent medium. The inhibitory compounds produced by the *Pseudomonas* and *Pseudoalteromonas* isolates were resistant to proteolytic enzymes (trypsin and pepsin) and sensitive to heat treatment. Inhibitory compounds produced by isolate P8 were also resistant to proteolytic enzymes and the activity was only lost after heat treatment for 5 min at 100°C.

In summary, the five isolates P8, P9, P11, P12, and P20, isolated from the surface of *Padina pavonica* and identified as closely related to *B. pumilus, Pseudomonas* sp., and *Pseudoalteromonas* produce bioactive compounds that act against human pathogens such as *S. aureus;* the most pathogenic specie of the genus *Staphylococcus* and of which multi-resistant strains represent a major problem in hospital (Le Loir et al., 2003). They also act against *V. alginolyticus*, which is one of the major causes of bacterial infections on shrimp farms (Hill et al., 2009), and against the virulent Gram- bacterium *V. vulnificus* causing gastroenteritis, necrotic skin infections, meningitis and fatality (Mayer et al., 2014), as well as against a number of fish pathogens. Given the broad range of pathogens that are inhibited, these isolates may produce compounds that might prove promising candidates for potentially novel antibiotic and antifungal drugs.

## Author contributions

AI performed all the experiments, analyzed and interpreted data, wrote and revised the manuscript. LK contributed in designation of the research plan, organization of the study, the development of work and corrected and evaluated the manuscript. MH assisted the molecular manipulations and sequencing, contributed to the blast, phylogentic analysis. HB was involved in design of the experiments, evaluation of outcome and involved in manuscript writing. MC did the *Vibrio* strains identification, contributed to data analyzes, interpretation and manuscript final finalization. AB and LS revised critically the manuscript and approved final version to be published. ME designed the research plan, organized and supervised the development of work, corrected and evaluated the manuscript, submitted the full sequences for assignment in GENBANK and supervised the whole work.

## Funding

This work was co-funded and supported by the two projects “MOTOX” and “BIOMER” from the National Institute of Marine Sciences and Technologies (INSTM).

### Conflict of interest statement

The authors declare that the research was conducted in the absence of any commercial or financial relationships that could be construed as a potential conflict of interest.
